# Parenting stress and health-related quality of life among parents of extremely preterm born early adolescents in England: a cross-sectional study

**DOI:** 10.1136/archdischild-2023-325429

**Published:** 2024-04-18

**Authors:** Emmi Suonpera, Anne Lanceley, Yanyan Ni, Neil Marlow

**Affiliations:** 1 EGA UCL Institute for Women's Health, University College London, London, UK; 2 School of Public Health, LKS Faculty of Medicine, The University of Hong Kong, Hong Kong, Hong Kong

**Keywords:** Adolescent Health, Intensive Care Units, Neonatal, Mental health, Neonatology

## Abstract

**Objective:**

To determine whether extremely preterm (EP) birth exerts persisting effects on parents in early adolescence.

**Design:**

Cross-sectional survey conducted between March 2017 and October 2018.

**Setting:**

Evaluation of a longitudinal population-based birth cohort in England at 11 years of age (EPICure2@11 Study).

**Participants:**

Parents of EP (<27 weeks of gestation) adolescents and control parents of term born (≥37 weeks of gestation) classmates of similar age and sex.

**Main outcome measures:**

The Parenting Stress Index Short Form (PSI-4-SF) and the Short Form Health Survey (SF-12v1).

**Results:**

The 163 EP and 125 comparison respondents were most commonly mothers in their mid-40s. EP parents reported higher total parenting stress scores compared with controls, overall (adjusted difference in means: 14 (95% CI 9 to 20)) and after exclusion of moderate and severe child disability and multiples (9 (95% CI 3 to 15). Average physical and mental health-related quality of life scores were similar in the two groups (adjusted difference in means physical health: −2 (95% CI −4 to 1) and mental health: −1 (95% CI −4 to 1)). Among EP parents, 12% (20/164) reported the combination of high parenting stress and low mental health scores. With increasing child age, parenting stress scores for preterm parents were lower in contrast to controls who reported increasing parenting stress.

**Conclusions:**

In early adolescence, compared with parents of term-born children, EP parents experience increased levels of parenting stress that are particularly high among a proportion of parents and associated with lower mental health-related quality of life. Practitioner awareness of this continuing risk throughout childhood is important to support parental abilities and well-being.

**WHAT IS ALREADY KNOWN ON THIS TOPIC**

- Stress and mental health problems among parents following preterm birth are well documented, particularly among mothers.
- Despite recent interest in parent outcomes following preterm birth, most studies focus on the early years.

**WHAT THIS STUDY ADDS**

- At around 11 years of age, parents of term-born children, English parents of extremely preterm children experienced increased levels of parenting stress. Parenting stress scores were particularly high among a proportion of parents and associated with low mental health-related quality of life.

**HOW THIS STUDY MIGHT AFFECT RESEARCH, PRACTICE OR POLICY**

- Practitioner awareness of continued adverse effects among the parents relating to their adolescent child’s extreme prematurity is important to support parental abilities and well-being.

## Introduction

Parents of children with long-term morbidities, particularly mothers, report increased stress and decreased psychological health relating to their roles as parents impacted by their child’s complex care needs.^1 2^ Preterm birth is associated with long-term behavioural, developmental and chronic health issues,^3^ with the highest prevalence among extremely preterm (EP) births (<28 weeks of gestation).^4^ Psychological distress among parents following preterm birth is well documented^5^ and may impact the parent–child relationship, parenting behaviour, child development and parental well-being.^6 7^

Despite recent interest in parent outcomes following preterm birth,^8^ most studies focus on the early years following EP birth and hospitalisation. At these times, the influence of the perinatal experience may still be active, and these studies do not reveal how parents’ experiences change over time.^9^ Parenting stress has rarely been examined later in adolescence.^10 11^ By early adolescence, parents have cumulative experience of the parent–child relationship and increased parental knowledge of their child’s health profile, but new cognitive, physical and social challenges appear as increased independent functioning and peer relations develop. EP parents may encounter additional stressors while supporting their child’s transition into adulthood.^12^ Understanding the parental impact during this period is crucial for effective support, improving parental well-being and optimising adolescent outcomes.

We have evaluated parenting stress and health-related quality of life (HRQoL) outcomes among the primary caregivers of EP and full-term born (≥37 weeks of gestation) individuals in early adolescence by using cross-sectional survey data collected as part of the evaluation of a longitudinal national birth cohort study of extreme prematurity in England (the EPICure2@11 Study). Based on literature, we hypothesised that caring for an early adolescent with neurodevelopmental impairments (NDIs) contributes towards increased parenting stress among EP parents.

## Methods

This study used data from the EPICure2 cohort, a national study of 1041 infants born in England <27 weeks of gestation in 2006 who were discharged alive from hospital. The cohort’s earlier phases^13^ and outcomes relating to the children at 2.5^14^ and at 11 years of age^15^ have been published.

Between March 2017 and October 2018, cohort children from two purposefully selected geographical areas in England based on study centres in London and Leicester were invited to take part in a school-based or home-based clinical child assessment of neurodevelopmental functioning.^15^ The selected neonatal networks were representative of the total EPICure2 cohort as per infant and child survival rate, maternal Index of Multiple Deprivation (IMD)^16^ at birth and proportion of children assessed at 2.5 years. With the help of head teachers, full-term born (≥37 weeks of gestation) classmates of similar age (±3 months) and sex were recruited as controls. No controls were recruited for children attending special educational needs and disabilities (SEND) schools or units.

One parent from each family who self-identified as the primary caregiver of the study participant/s was invited to complete a postal questionnaire comprising the Parenting Stress Index-Short Form (PSI-4-SF),^17^ the Short Form Health Survey (SF-12v1)^18^ and sociodemographic data.

The a priori primary outcome measure, PSI-4-SF, is a well-validated 36-item self-report questionnaire assessing the level of stress in the parent–child system in three 12-item domains of parental distress (PD), parent–child dysfunctional interaction (P-CDI) and difficult child (DC) derived from a 5-point Likert scale response to each statement, rated from ‘strongly agree’ to ‘strongly disagree’ (higher scores indicating more stress).^19 20^ The three domains are summated as a Total Stress Score (TSS). A TSS above 109 (>84th percentile) is published clinical cut-off for high parenting stress.^17 21 22^ A further 6-item Defensive Responding (DR) scale is measured. Indicating internal consistency, Cronbach’s alpha (α) was high in each domain and total scores by the groups of parents (PD 0.91 and 0.87; P-CDI 0.87 and 0.89; DC 0.92 and 0.88; TSS 0.96 and 0.94; DR 0.86 and 0.80 for EP and control parents, respectively).

Parents also completed the SF-12v1 instrument which was scored as recommended to derive perceived general physical (PCS-12) and mental (MCS-12) HRQoL in the past 4 weeks.^23^ Both the PCS-12 and the MCS-12 composite scores range from 0 to 100; higher scores indicating better HRQoL. The standardised scoring for SF-12v1 allows direct comparisons to general populations. Scores >50 are above average, and each 1-point score increase represents a 0.1 SD.^24^ Internal consistency (α) by the groups of parents was high (EP parents: 0.87; controls: 0.78).

Parents were asked about their age, ethnicity, employment status, marital status, living arrangements, other children, education, receiving of governmental income support and residential postcode to determine the family’s IMD score as an indication of socioeconomic status. The IMD is a relative small-area measure of deprivation in England categorised into deciles (1/10) based on the English population (lowest decile indicating highest deprivation).^16^

Child characteristics included sex, age, school type, multiplicity (index children only) and presence of NDI categorised as none/mild, moderate or severe (defined based on assessments of cognition, manual ability, gross-motor functioning, vision and hearing)^15^ collected during the clinical evaluation of the child.

Analyses were performed at the parent level, except for the PSI-4-SF which is examined per parent–child dyad. Appropriate univariate analyses were performed to assess potential group differences between EP and control parents. Group comparisons between EP and control parents were performed for each outcome measure adjusting for child’s male sex and age, parent age, ethnicity and IMD decile at the time of the child assessment. Family was added as a random effect to adjust for within family clustering of outcomes when applicable. Parents with missing responses were excluded from the adjusted analyses. Group differences were presented as adjusted differences in means together with 95% CIs. To account for multiple testing, Bonferroni correction was applied. The group differences were tested by excluding the parents with children with moderate or severe NDI and/or multiples.

To assess factors associated with high TSS among EP parents, logistic regression analysis was performed to examine associations of sociodemographic factors (ie, child age, school type, receipt of income support) and child disability level with TSS as supported by literature.^1 25^ Variables associated with high TSS in the univariate analyses (p≤0.10) were included in the multivariable logistic regression models, except HRQoL. As HRQoL and PSI correlated with other predictors in the same model, HRQoL was excluded from the adjusted model. To avoid small cell counts (<10), school type and child disability level were dichotomised. ORs were reported with 95% CI. On all analyses, a priori p value of <0.05 was considered significant. Analyses were performed using STATA, V.17.0 (StataCorp LLC).

## Results

**Figure 1:**
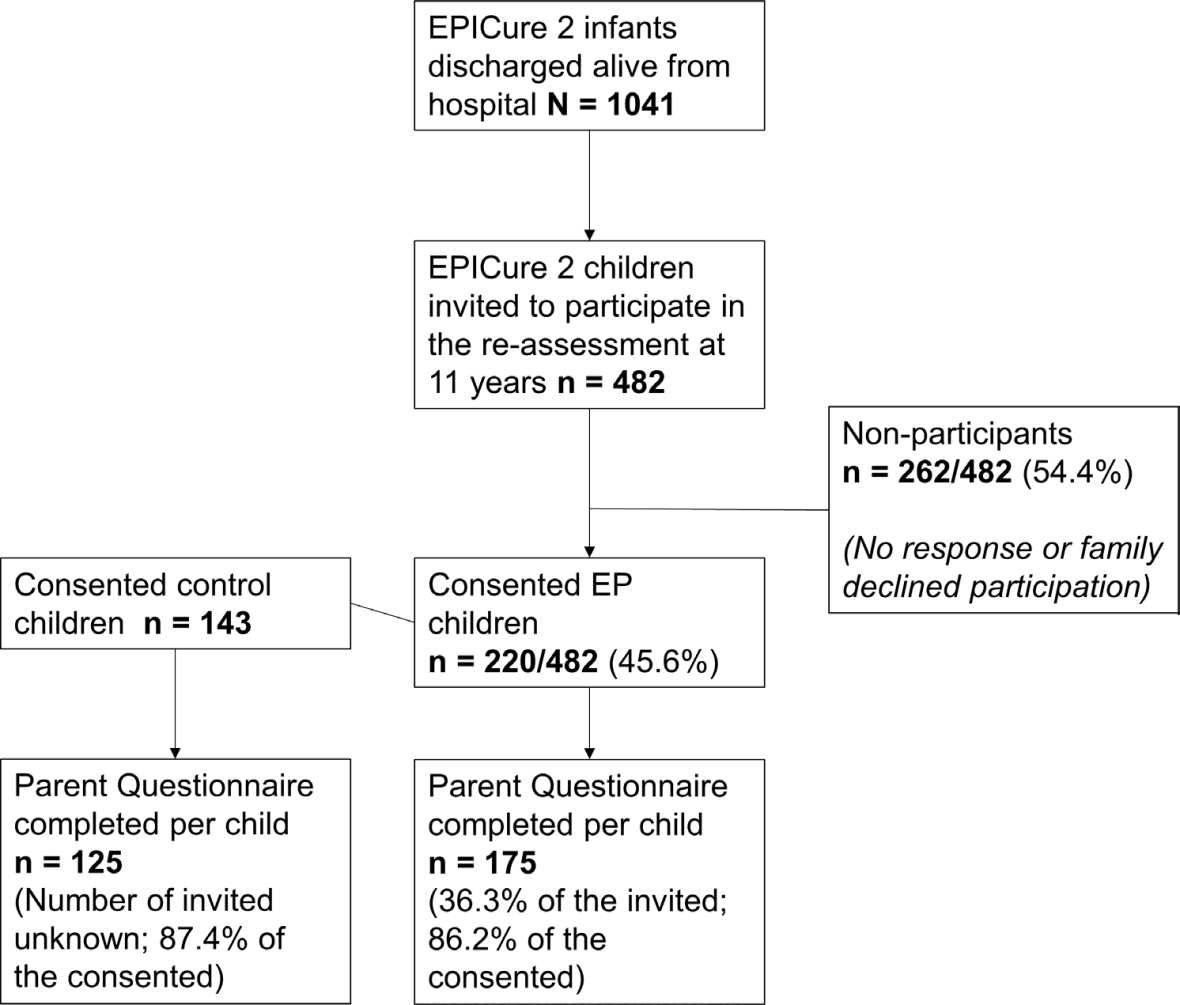
Flowchart of the participants in the present study. EP, extremely preterm.

Participants were 163 parents of 175 EP children (including 10 parents with twins and one parent with triplets) and 125 control parents who consented for their child to participate in the EPICure2@11 Study at around 12 years (age range: 10.4–13.0 years). Of the 482 invited EP children (46.3% (482/1041) of the total EPICure2 cohort) (figure 1), parents consented for 220 children to be clinically assessed, and parents completed questionnaires for 175 of these children (36.3% of those invited; 86.2% of those consented) (figure 1). Of the 143 control children whose parents consented to take part in the EPICure@11 Study, 125 completed their questionnaires (response rate 87.4%). As schools recruited the control families, refusal rates were not available.

Comparable perinatal characteristics between cohort children assessed at 11 years and non-participants have been reported previously.^15^ The evaluated sample of children had similar distribution of gestational age, men and multiples but heavier birthweights compared with those who consented but were unevaluated. Respondents were more often from white ethnic backgrounds and had on average higher IMD deciles at birth (online supplemental table 1).

10.1136/fetalneonatal-2023-325429.supp1 Supplementary data

Among both groups, the respondents were most commonly mothers in their mid-40s, employed and married or living with the other parent of the study child (table 1) and 43% had a university degree (115/265). EP parents were more commonly non-white (37% vs 19%; p=0.008) or received governmental income support (63% vs 48%; p=0.01). EP and control children characteristics were of similar ages and sex distribution. Missing responses were uncommon (<10% per variable; please see tables 1 and 2 for number of responses per variable).

**Table 1:** Characteristics of parents and children included in analyses

	Extremely preterm (n=163)	Control (n=125)	P value*
**Parent characteristics**			
Respondent (n=280)			
Mother	144 (91%)	109 (90%)	0.69
Father	12 (7%)	11 (9%)	
Carer/foster parent/grandparent/friend	3 (2%)	1 (1%)	
Median parental age in years (n=278)	46 (IQR 42–50)	47 (IQR 42–49)	0.72
Employment status (n=285)			
Employed/self-employed/full-time student	124 (76%)	101 (82%)	0.52
Retired/semi-retired/long-term illness	4 (3%)	2 (2%)	
Homemaker/carer	23 (14%)	16 (13%)	
Unemployed	11 (7%)	4 (3%)	
Marital status (n=287)			
Married/living with a partner	125 (77%)	99 (79%)	0.14
Separated/divorced/widowed	15 (9%)	17 (14%)	
Single	22 (14%)	9 (7%)	
Living with the father or mother of the study child (n=287)	116 (72%)	95 (76%)	0.40
Highest academic qualification (n=265)			
University degree	60 (40%)	55 (47%)	0.51
Some post-secondary education	44 (30%)	30 (26%)	
Secondary education or less	45 (30%)	31 (27%)	
Ethnicity (n=288)			
White	103 (63%)	101 (81%)	**0.006**
Mixed/multiple/other ethnic groups	12 (7%)	4 (3%)	
Asian/Asian British	25 (15%)	14 (11%)	
Black/African/Caribbean/black British	23 (14%)	6 (5%)	
Receiving family and/or income support or tax credits (n=288)	102 (63%)	60 (48%)	**0.01**
Average IMD at 11 years (n=277)†	5.5 (2.8 SD)	5.6 (2.9 SD)	0.60
Average IMD at delivery (n=158)‡	4.7 (2.7 SD)	n/a	
**Child characteristics**§			
Male sex (n=297)	90 (52%)	55 (44%)	0.16
Average age in years (n=297)	12 (0.6 SD)	12 (0.6 SD)	0.26
Median gestational age in weeks (n=172)	26 (range 23–26.9)	n/a	
Average number of siblings (n=294)	1 (0.9 SD)	1 (1.0 SD)	0.18
School type (n=296)			
Mainstream	148 (86%)	124 (100%)	n/a
Special educational needs school or unit	21 (12%)		
Home educated	3 (2%)		
Overall neurodevelopmental disability (n=297)			
None or mild	109 (63%)	121 (97%)	n/a
Moderate	33 (19%)	4 (3%)	
Severe	30 (18%)	0 (0%)	
Multiple status at birth (n=172)	43 (25%)	–	–

Note: Missing data accounts for differing totals.

Bold p value indicates a priori defined statistical significance (p<0.05).

*χ^2^ test or Fisher’s exact test for categorical variables and Welch’s t-test for continuous variables were used.

†Parents resident in England at the time of the child re-assessment at 11 years. EP n=156 and control n=121, respectively.

‡Biological parents who returned Parent Questionnaires at 11 years (one missing postcode at birth and four non-biological carers at 11 years).

§Children who were clinically re-assessed at 11 years and Parent Questionnaires were returned. EP n=172 and control n=125, respectively.

EP, extremely preterm; IMD, Index of Multiple Deprivation.

**Table 2:** Parenting stress and health-related quality of life scores and rates of respondents reporting high levels of stress

	Extremely preterm (n=175)*	Control (n=125)	P value†
Average parenting stress PSI-4-SF score			
Total Stress Score (n=291)	74.1 (26.3 SD)	59.3 (17.0 SD)	**<0.001**
Parental distress (n=295)	24.2 (9.8 SD)	21.0 (6.9 SD)	**0.04**‡
Parent–child dysfunctional interaction (n=294)	23.4 (8.6 SD)	18.6 (6.3 SD)	**<0.001**‡
Difficult child (n=295)	26.6 (10.7 SD)	19.7 (6.3 SD)	**<0.001**‡
Parent reporting high parenting stress§			
Total stress (n=291)	20 (12%)	0	**<0.001**
Parental distress (n=295)	20 (12%)	2 (2%)	**0.003**‡
Parent–child dysfunctional interaction (n=294)	23 (14%)	3 (2%)	**0.003**‡
Difficult child (n=295)	30 (18%)	3 (2%)	**0.003**‡
Defensive responding (n=296)	52 (30%)	47 (38%)	0.17

	Extremely preterm (n=163)	Control (n=125)	P value†
Average parental health-related quality of life SF-12v1 score			
Physical health (n=276)	50.9 (9.2 SD)	52.3 (8.0 SD)	0.16
Mental health (n=276)	49.2 (9.5 SD)	50.7 (8.1 SD)	0.16

Note: Missing data accounts for differing totals.

Bold p value indicates a priori defined statistical significance (p<0.05).

*All returned Parent Questionnaires at 11 years (child re-assessed at 11 years and Parent Questionnaire returned n=172; Parent Questionnaire returned but child not assessed at 11 years n=3).

†χ^2^ test or Fisher’s exact test for categorical variables and Welch’s t-test for continuous variables were used.

‡Bonferroni correction (p values are multiplied by 3).

§Raw scores Total Stress Score >109, parental distress >37, parent–child dysfunctional interaction >33, difficult child >37 indicate high stress, Defensive Responding Raw scores ≤10 is suggestive of defensive responding.^17^

IMD, Index of Multiple Deprivation^16^; PSI-4-SF, Parenting Stress Index-Short Form fourth Edition; SF-12v1, Short Form Health Survey version 1.

Parents of EP children reported higher levels of parenting stress overall and across all domains compared with controls (table 2). The mean TSS among EP parents was 74 compared with 59 among controls (adjusted difference in means: 14 (95% CI 9 to 20; p<0.001)) (table 3). Using the published clinical cut-off for high TSS,^17^ 12% of EP parents (20/175) were categorised as having high stress compared with none of the control parents (0/125) (p<0.001) and was reflected in higher proportions within each subscale score among the EP group. The Defensive Reporting scale showed similar values in EP and control parents (30% vs 38%, respectively; p=0.17; table 2).

**Table 3:** Adjusted differences in means in average PSI-4-SF and SF-12v1 scores between the groups of parents

	n/N*	Unadjusted sample mean	(95% CI)	Adjusted† sample mean	(95% CI)	Adjusted† difference in means	(95% CI)	P value‡
PSI-4-SF								
Total stress								
EP	158/175	73.8	(69.6 to 77.9)	73.6	(69.9 to 77.2)	14.2	(8.5 to 19.9)	**<0.001**
Control	112/125	58.6	(55.4 to 61.8)	59.4	(55.1 to 63.6)			
Parental distress								
EP	161/175	24.1	(22.6 to 25.6)	23.9	(22.5 to 25.2)	2.8	(0.7 to 5.0)	**0.03**
Control	112/125	20.9	(19.6 to 22.2)	21.0	(19.4 to 22.6)			
Parent–child dysfunctional interaction								
EP	161/175	23.3	(22.0 to 24.7)	23.3	(22.0 to 24.5)	4.6	(2.7 to 6.5)	**<0.001**
Control	112/125	18.3	(17.2 to 19.4)	18.6	(17.2 to 20.0)			
Difficult child								
EP	161/175	26.4	(24.7 to 28.0)	26.3	(24.9 to 27.8)	6.8	(4.5 to 9.0)	**<0.001**
Control	112/125	19.4	(18.2 to 20.6)	19.6	(17.9 to 21.3)			
Total stress								
EP	93/97	67.2	(61.9 to 72.4)	67.6	(63.2 to 72.1)	8.6	(2.5 to 14.8)	**0.006**
Control	109/121	58.5	(55.2 to 61.7)	58.9	(54.8 to 63.0)			
Parental distress								
EP	94/97	21.8	(20.0 to 23.6)	21.7	(20.1 to 23.4)	0.8	(–1.5 to 3.1)	>0.99
Control	109/121	20.9	(19.5 to 22.3)	21.0	(19.4 to 22.5)			
Parent–child dysfunctional interaction								
EP	93/97	21.4	(19.5 to 23.2)	21.5	(19.9 to 23.0)	3.0	(0.8 to 5.1)	**0.021**
Control	109/121	18.3	(17.1 to 19.4)	18.5	(17.0 to 19.9)			
Difficult child								
EP	94/97	24.2	(22.0 to 26.3)	24.4	(22.7 to 26.2)	5.0	(2.6 to 7.5)	**<0.001**
Control	109/121	19.3	(18.1 to 20.5)	19.4	(17.7 to 21.0)			
SF-12v1								
Physical Component Summary score (PCS-12)								
EP	148/163	50.8	(49.3 to 52.3)	51.0	(49.6 to 52.4)	1.5	(–3.7 to 0.7)	0.18
Control	107/125	52.4	(50.8 to 53.9)	52.4	(50.8 to 54.1)			
Mental Component Summary score (MCS-12)								
EP	148/163	49.4	(47.8 to 50.9)	49.3	(47.9 to 50.7)	1.3	(–3.5 to 0.9)	0.24
Control	107/125	50.7	(49.2 to 52.2)	50.6	(48.9 to 52.3)			
SF-12v1: restricted to singletons with no/mild impairment								
Physical Component Summary score (PCS-12)								
EP	91/97	50.9	(48.9 to 53.0)	50.8	(49.0 to 52.6)	1.9	(–4.4 to 0.6)	0.14
Control	104/121	52.3	(50.7 to 53.9)	52.7	(51.0 to 54.4)			
Mental Component Summary score (MCS-12)								
EP	91/97	51.4	(49.8 to 53.1)	51.1	(49.5 to 52.8)	0.6	(–1.6 to 2.9)	0.57
Control	104/121	50.8	(49.2 to 52.3)	50.5	(49.0 to 52.0)			

Bold p value indicates a priori defined statistical significance (p<0.05).

*Those parents with complete set of data/all parents who returned their Parent Questionnaires at 11 years.

†Adjusted for child age, child male sex, parent age, parent ethnicity, the Index of Multiple Deprivation^16^ decile at 11 years, interaction of child age and birth status if present and family as a random effect if applicable.

‡Multiple linear regression with Bonferroni correction (p values multiplied by 3; Total Stress Score not corrected).

EP, extremely preterm; PSI-4-SF, Parenting Stress Index-Short Form 4th Edition.

After excluding parents of children with moderate/severe NDI and non-singletons, mean TSS reduced but remained higher in EP parents (adjusted difference: 9 points, 95% CI 3 to 15; p=0.006) and was reflected in higher subscale scores for parent–child dysfunctional interaction and difficult child. EP parents reported highest subscale scores in difficult child, even after excluding adolescents with NDI and multiples (table 3).

In contrast to PSI results, SF-12v1 scores were similar in both physical and mental health domains between groups and remained similar after exclusion of children with moderate/severe impairment/multiplicity (table 3).

**Figure 2:**
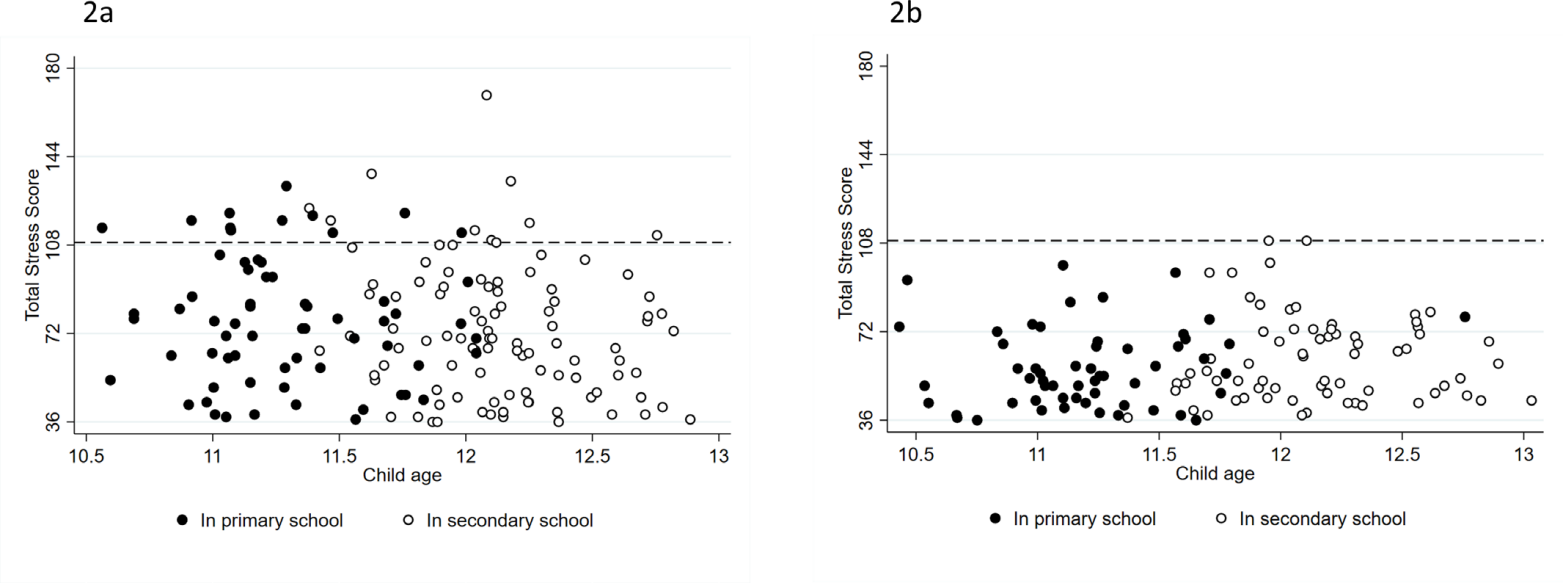
Scatter plot comparing PSI-4-SF Total Stress scores by child’s age in years among parents of extremely preterm born early adolescents (A) and control parents (B) whose children were in primary school and those who were in secondary school. Dots represent Total Stress Scores per child among respondents whose child underwent a clinical child assessment. The dashed line represents the cut-off for high parenting stress (>84th centile; raw score >109).^17^ (A) Extremely preterm children in primary school n=65 and in secondary school n=99. (B) Control children in primary school n=56 and in secondary school n=68.

The child’s age was associated with TSS differently within the two groups of parents; among EP parents total scores were higher in those with younger children, in controls scores were higher in older children. Introduction of an interaction factor (child age/birth status) improved the fit of the model significantly (χ^2^(1)=5.39, p=0.02). A similar pattern was seen in the parent–child dysfunctional interaction and difficult child domains, but not in parental distress. Transition to secondary education occurs in early adolescence, but similar proportions of parents reported high TSS pre and post child transition among the respondents (figure 2).

In univariate analyses, receipt of income support, presence of moderate/severe NDI, need for special educational needs (SEN) schooling and younger child age were associated with high TSS. There were no statistically significant associations between high TSS and parent sociodemographic characteristics.

In the multivariable model, only the association between child attending SEN and high TSS remained significant (Adjusted Odds Ratio 4, 95% CI 1 to 15; p=0.03) (table 4).

**Table 4:** Demographic and health variables associated with high total parenting stress scores (PSI-4-SF) among parents of extremely preterm born early adolescents: n, row %, unadjusted and multivariable logistic regression

Demographic and health variables	Proportion of parents reporting high Total Stress Scores 20/164* (12.2%)	OR	(95% CI)	P value	AOR†	(95% CI)	P value
Physical Component Summary score (PCS-12)	45.2 (11.9 SD)/51.5 (8.7 SD)	0.9	(0.9 to 1.0)	**0.01**			
Mental Component Summary score (MCS-12)	37.5 (12.4 SD)/51.2 (7.4 SD)	0.9	(0.8 to 0.9)	**<0.001**			
Receipt of income support							
No	4/67 (6.0%)	1.0			1.0		
Yes	16/97 (16.5%)	3.1	(1.0 to 9.8)	**0.05**	2.5	(0.7 to 8.1)	0.14
Child age‡	11.6 (0.5 SD)/11.8 (0.5 SD)	0.4	(0.2 to 1.0)	**0.06**	0.4	(0.1 to 1.0)	0.06
School type							
Mainstream or home educated	13/145 (9.0%)	1.0			1.0		
SEN school or unit	7/19 (36.8%)	5.9	(2.0 to 17.7)	**0.001**	4.1	(1.1 to 15.4)	**0.03**
Moderate/severe neurodevelopmental disability							
No	8/107 (7.5)	1.0			1.0		
Yes	12/57 (21.1)	3.3	(1.3 to 8.6)	**0.02**	1.6	(0.5 to 5.1)	0.43

Note: High Total Stress Scores (TSS) defined as PSI-4-SF scores >84th centile.^17^

Bold p value indicates a priori defined statistical significance (p<0.05).

*Those who returned their parent questionnaire, whose child underwent clinical child assessment and TSS was not missing or incomplete.

†Adjusted for all other variables associated with high total parenting stress in univariate analyses (p<0.10).

‡Reported as mean (SD) among high stress parents/mean (SD) among low stress parents.

## Discussion

In adolescence, regardless of the presence of NDI, EP parents reported higher levels of parenting stress in comparison with parents of classmates after adjusting for parent and child age, male sex, and family’s IMD, and parent ethnicity. Previous longitudinal evidence indicates a stable decline in stress among parents of very preterm infants (28–32 weeks of gestation) over time,^25^ although distress scores may plateau in adolescence.^26^ In our study of extremely immature infants, EP parents reported similar TSS to parents of young infants; a meta-analysis of parenting stress among parents of preterm born children (mean gestational age 32 weeks) reported a pooled average TSS of 71.6 (95% CI, 68.3 to 74.8) from five studies utilising the PSI-SF instrument at 0–18 months after the birth.^27^

Although elevated, the mean TSS reported by EP parents fell within the accepted normal range. This is consistent with research on younger very preterm populations.^11 27–30^ The generally high SES^31^ and older parent age^30^ in our sample may have contributed towards lower scores, although some evidence suggests that older parents with high family resources and educational attainment may experience increased stress.^12 32 33^ A larger proportion of EP parents in this study were from minority ethnic backgrounds, which has been associated with poorer parent outcomes.^34^ Some level of stress is typical in any parent–child relationship,^35^ and the excess parenting stress reported by EP parents may be attributed to their child’s health concerns and/or developmental delays.^15^

Contrary to our hypothesis, heightened parenting stress could not be predominantly attributed to the presence of child NDI. Higher average scores persisted in all domains, except in parental distress, after excluding parents of early adolescents with impairments and non-singletons. The measure of child disability utilised in this study focused on neurodevelopmental factors and did not include child behavioural or psychiatric measures, which may partially explain this finding.^1^ This interpretation is supported by the finding that SEND schooling had a stronger association with high TSS than child disability. Studies among parents of adolescents from other clinical populations suggest that behavioural and social challenges in children have a stronger association with high parenting stress than physical disability or severe illness.^10^ If parenting stress among EP parents is triggered by their child’s behavioural traits, the reduction in parent–child interactional domain scores with increasing child age is a reassuring finding.

The contrasting effects of child age on parent–child dysfunctional outcomes among EP and control parents have been reported before.^25^ EP families may experience reduced or delayed adolescence-related family conflict compared with controls,^36 37^ which may in part explain the diverging trends. As reduced family conflict may be associated with increased parental control and delayed adolescent independence,^37^ parental support to facilitate EP child’s transition to increased independence in adolescence is important.

The increased level of parental distress among EP parents compared with controls diminished when parents of adolescents with severe/moderate NDI and multiples were excluded. The child’s age did not have a differential impact on this outcome between the groups, suggesting that personal distress in the parenting role is influenced by the presence of child disability and/or multiplicity rather than the child’s age or birth status. The association between high TSS and low mental HRQoL among EP parents suggests that the increased psychological ill health experienced by EP parents with children with NDI is related to their parenting roles. Studies have reported a correlation between parental psychological ill health and increased parenting stress.^29 38^ Maternal mental health outcomes in the EPICure2 cohort have not been examined previously, which limits our understanding of pre-existing conditions.

The present study had limitations. Although response rates among consented parents were high in both groups of parents (>80%), the sample of parents taking part showed a high attrition rate. Only approximately 40% of the approached families in the EPICure2 cohort provided parenting stress data. It is not possible to estimate the impact of non-respondents’ stress scores on the overall results. Neonatal outcomes of respondents and non-respondents were similar in male sex and multiplicity. Yet, parents who completed the questionnaire were more likely to be from white ethnic backgrounds and had higher SES, factors that have been associated with low^1^ and heightened parenting stress.^25^ Non-response bias resulting from lost to follow-up of families from higher sociodemographic risk groups is a frequent challenge of longitudinal cohort studies.^39 40^ Additionally, the challenge of recruiting control participants from SEND settings should be acknowledged. This may have in part explained higher TSS among EP parents, although excluding adolescents with moderate/severe NDI did not alter the overall results.

In conclusion, extreme prematurity is still associated with parenting stress a decade after the child’s birth. Although the stress scores fell within the normal range, EP parents reported increased levels of parenting stress across all stress domains independent of child NDI. Given the proportion of EP parents reporting high parenting stress and low mental HRQoL, practitioners should be aware of this continuing risk to support parental abilities and well-being.

## Data Availability

Data are available upon reasonable request. Data are available upon reasonable request. Data are available subject to the EPICure Data Sharing Policy (http://www.epicure.ac.uk http://www.epicure.ac.uk).

## References

[1] Almogbel YS , Goyal R , Sansgiry SS . Association between parenting stress and functional impairment among children diagnosed with neurodevelopmental disorders. Community Ment Health J 2017;53:405–14. 10.1007/s10597-017-0096-9 28176211

[2] Wiener J , Biondic D , Grimbos T , et al . Parenting stress of parents of adolescents with attention-deficit hyperactivity disorder. J Abnorm Child Psychol 2016;44:561–74. 10.1007/s10802-015-0050-7 26183609

[3] Johnson S , Marlow N . Early and long-term outcome of infants born extremely preterm. Arch Dis Child 2017;102:97–102. 10.1136/archdischild-2015-309581 27512082

[4] Pierrat V , Marchand-Martin L , Arnaud C , et al . Neurodevelopmental outcome at 2 years for preterm children born at 22 to 34 weeks' gestation in France in 2011: EPIPAGE-2 cohort study. BMJ 2017;358:j3448. 10.1136/bmj.j3448 28814566 PMC5558213

[5] Caporali C , Pisoni C , Gasparini L , et al . A global perspective on parental stress in the neonatal intensive care unit: a meta-analytic study. J Perinatol 2020;40:1739–52. 10.1038/s41372-020-00798-6 32901116

[6] Forcada-Guex M , Borghini A , Pierrehumbert B , et al . Prematurity, maternal posttraumatic stress and consequences on the mother-infant relationship. Early Hum Dev 2011;87:21–6. 10.1016/j.earlhumdev.2010.09.006 20951514

[7] Wilson C , Cook C . Ambiguous loss and post-traumatic growth: experiences of mothers whose school-aged children were born extremely prematurely. J Clin Nurs 2018;27:e1627–39. 10.1111/jocn.14319 29495088

[8] Zeitlin J , Sentenac M , Morgan AS , et al . Priorities for collaborative research using very preterm birth cohorts. Arch Dis Child Fetal Neonatal Ed 2020;105:538–44. 10.1136/archdischild-2019-317991 32029530 PMC7547907

[9] Janvier A , Lantos J , Aschner J , et al . Stronger and more vulnerable: a balanced view of the impacts of the NICU experience on parents. Pediatrics 2016;138:e20160655. 10.1542/peds.2016-0655 27489297

[10] Barroso NE , Mendez L , Graziano PA , et al . Parenting stress through the lens of different clinical groups: a systematic review & meta-analysis. J Abnorm Child Psychol 2018;46:449–61. 10.1007/s10802-017-0313-6 28555335 PMC5725271

[11] Treyvaud K . Parent and family outcomes following very preterm or very low birth weight birth: a review. Semin Fetal Neonatal Med 2014;19:131–5. 10.1016/j.siny.2013.10.008 24252709

[12] Moore M , Gerry Taylor H , Klein N , et al . Longitudinal changes in family outcomes of very low birth weight. J Pediatr Psychol 2006;31:1024–35. 10.1093/jpepsy/jsj075 16150877

[13] Costeloe KL , Hennessy EM , Haider S , et al . Short term outcomes after extreme preterm birth in England: comparison of two birth cohorts in 1995 and 2006 (the epicure studies). BMJ 2012;345:e7976. 10.1136/bmj.e7976 23212881 PMC3514472

[14] Moore T , Hennessy EM , Myles J , et al . Neurological and developmental outcome in extremely preterm children born in England in 1995 and 2006: the epicure studies. BMJ 2012;345:e7961. 10.1136/bmj.e7961 23212880 PMC3514471

[15] Marlow N , Ni Y , Lancaster R , et al . No change in neurodevelopment at 11 years after extremely preterm birth. Arch Dis Child Fetal Neonatal Ed 2021;106:418–24. 10.1136/archdischild-2020-320650 33504573

[16] Ministry of Housing Communities and Local Government . The English Indices of Deprivation 2019 Research report. London: The Crown, 2019.

[17] Abidin RR . Parenting Stress Index Fourth Edition Professional Manual. Lutz FL: PAR, 2012.

[18] Jenkinson C , Layte R . Development and testing of the UK SF-12. J Health Serv Res Policy 1997;2:14–8. 10.1177/135581969700200105 10180648

[19] Barroso NE , Hungerford GM , Garcia D , et al . Psychometric properties of the parenting stress index-short form (PSI-SF) in a high-risk sample of mothers and their infants. Psychol Assess 2016;28:1331–5. 10.1037/pas0000257 26595220 PMC4877285

[20] Touchèque M , Etienne A , Stassart C , et al . Validation of the French version of the parenting stress index-short form. J Community Psychol 2016;44:419–25. 10.1002/jcop.21778

[21] Carotenuto M , Esposito M , Di Pasquale F , et al . Psychological, cognitive and maternal stress assessment in children with primary Ciliary dyskinesia. World J Pediatr 2013;9:312–7. 10.1007/s12519-013-0441-1 24235065

[22] Esposito M , Marotta R , Roccella M , et al . Pediatric neurofibromatosis 1 and parental stress: a multicenter study. Neuropsychiatr Dis Treat 2014;10:141–6. 10.2147/NDT.S55518 24489471 PMC3904813

[23] Gandek B , Ware JE , Aaronson NK , et al . Cross-validation of item selection and scoring for the SF-12 health survey in nine countries: results from the IQOLA project. J Clin Epidemiol 1998;51:1171–8. 10.1016/s0895-4356(98)00109-7 9817135

[24] Ware J , Kosinski M , Keller S . SF-12: how to score the SF-12 physical and mental health summary scales; 1998.

[25] Singer LT , Fulton S , Kirchner HL , et al . Longitudinal predictors of maternal stress and coping after very low-birth-weight birth. Arch Pediatr Adolesc Med 2010;164:518–24. 10.1001/archpediatrics.2010.81 20530301 PMC10222517

[26] Yaari M , Treyvaud K , Lee KJ , et al . Preterm birth and maternal mental health: longitudinal trajectories and predictors. J Pediatr Psychol 2019;44:736–47. 10.1093/jpepsy/jsz019 30977828 PMC7967874

[27] Brummelte S , Grunau RE , Synnes AR , et al . Declining cognitive development from 8 to 18 months in preterm children predicts persisting higher parenting stress. Early Hum Dev 2011;87:273–80. 10.1016/j.earlhumdev.2011.01.030 21334150

[28] Landsem IP , Handegård BH , Tunby J , et al . Early intervention program reduces stress in parents of preterms during childhood, a randomized controlled trial. Trials 2014;15:387. 10.1186/1745-6215-15-387 25282345 PMC4198672

[29] Linden MA , Cepeda IL , Synnes A , et al . Stress in parents of children born very preterm is predicted by child externalising behaviour and parent coping at age 7 years. Arch Dis Child 2015;100:554–8. 10.1136/archdischild-2014-307390 25762532

[30] Schappin R , Wijnroks L , Uniken Venema MMAT , et al . Rethinking stress in parents of preterm infants: a meta-analysis. PLoS One 2013;8:e54992. 10.1371/journal.pone.0054992 23405105 PMC3566126

[31] Singer LT , Fulton S , Kirchner HL , et al . Parenting very low birth weight children at school age: maternal stress and coping. J Pediatr 2007;151:463–9. 10.1016/j.jpeds.2007.04.012 17961686 PMC10228568

[32] Indredavik MS , Vik T , Heyerdahl S , et al . Low-birthweight adolescents: quality of life and parent-child relations. Acta Paediatr 2005;94:1295–302. 10.1111/j.1651-2227.2005.tb02091.x 16278996

[33] Jadva V , Lysons J , Imrie S , et al . An exploration of parental age in relation to parents' psychological health, child adjustment and experiences of being an older parent in families formed through egg donation. Reprod Biomed Online 2022;45:401–9. 10.1016/j.rbmo.2022.03.029 35644879 PMC10444692

[34] Holditch-Davis D , Miles MS , Weaver MA , et al . Patterns of distress in African-American mothers of preterm infants. J Dev Behav Pediatr 2009;30:193–205. 10.1097/DBP.0b013e3181a7ee53 19412125 PMC2755596

[35] Abidin RR . The determinants of parenting behavior. J Clin Child Psychol 1992;21:407–12. 10.1207/s15374424jccp2104_12

[36] Saigal S , Pinelli J , Streiner DL , et al . Impact of extreme prematurity on family functioning and maternal health 20 years later. Pediatrics 2010;126:e81–8. 10.1542/peds.2009-2527 20530081

[37] Burnett AC , Lee KJ , Cheong JLY , et al . Family functioning and mood and anxiety symptoms in adolescents born extremely preterm. J Dev Behav Pediatr 2017;38:39–48. 10.1097/DBP.0000000000000368 27984416

[38] Pinquart M . Parenting stress in caregivers of children with chronic physical condition-A meta-analysis. Stress Health 2018;34:197–207. 10.1002/smi.2780 28834111

[39] Gerstein ED , Poehlmann-Tynan J . Transactional processes in children born preterm: influences of mother-child interactions and parenting stress. J Fam Psychol 2015;29:777–87. 10.1037/fam0000119 26147934 PMC4743934

[40] Johnson S , Seaton SE , Manktelow BN , et al . Telephone interviews and online questionnaires can be used to improve neurodevelopmental follow-up rates. BMC Res Notes 2014;7:219. 10.1186/1756-0500-7-219 24716630 PMC3983863

