# Supplementary material for: Parenting stress and health-related quality of life among parents of extremely preterm born early adolescents in England: a cross-sectional study
Source: Arch Dis Child Fetal Neonatal Ed. 2024 Apr 18;109(3):253–60. doi: 10.1136/archdischild-2023-325429 (PMC11041583; doi:10.1136/archdischild-2023-325429)
Supplement: Supplementary data [file fetalneonatal-2023-325429supp001.pdf]

Supplementary Table 1 (Stable1). A comparison of characteristics at birth between those biological index children whose parents completed the parent questionnaire at 11 years (n=171) and mothers who consented for clinical child assessment at 11 years but failed to return the parent questionnaire (n=45)

**Characteristics**	**Parent Questionnaire completed n=171/216[a] (79%)**	**Parent Questionnaire not completed n=45/216[a] (21%)**	***P* value[b]**
**Infant male sex**	87 (51%)	19 (42%)	0.3
**Average gestational age in weeks**	26 (1 SD)	25 (1 SD)	0.2
**Average birthweight in grams**	822 (141 SD)	768 (149 SD)	**0.03**
**Multiple birth**	43 (25%)	7 (16%)	0.2
**Average IMD at delivery**	4.7 (2.6 SD)	3.6 (2.6 SD)	**0.02**
**Average age of mothers in years at birth**	32 (6 SD)	31 (6 SD)	0.7
**Maternal ethnicity**			
White	110 (64%)	14 (31%)	**<0.001**
Black/African/Caribbean/Black British	23 (13%)	17 (38%)	
Asian/Asian British	28 (16%)	12 (27%)	
Mixed and other ethnic groups	10 (6%)	2 (4%)	

[a] Characteristics at birth of 4 index children who were adopted or in foster care whose carers completed the parent questionnaire at 11 years were excluded
[b] Bolded *P* value indicates a priori defined statistical significance (*P* <0.05)
Chi Square Test for categorical variables and Welch's t-Test for continuous variables were used
IMD Index of Multiple Deprivation
SD Standard Deviation
